# Profiling walking dysfunction in multiple sclerosis: characterisation, classification and progression over time

**DOI:** 10.1038/s41598-018-22676-0

**Published:** 2018-03-21

**Authors:** Linard Filli, Tabea Sutter, Christopher S. Easthope, Tim Killeen, Christian Meyer, Katja Reuter, Lilla Lörincz, Marc Bolliger, Michael Weller, Armin Curt, Dominik Straumann, Michael Linnebank, Björn Zörner

**Affiliations:** 1 Department of Neurology, University Hospital and University of Zurich, Frauenklinikstrasse 26, 8091 Zurich, Switzerland; 2 Spinal Cord Injury Center, Balgrist University Hospital, Forchstrasse 340, 8008 Zurich, Switzerland; 3 Department of Neurology, Helios-Klinik Hagen-Ambrock, /University Witten/Herdecke, Ambrocker Weg 60, 58091 Hagen, Germany

## Abstract

Gait dysfunction is a common and relevant symptom in multiple sclerosis (MS). This study aimed to profile gait pathology in gait-impaired patients with MS using comprehensive 3D gait analysis and clinical walking tests. Thirty-seven patients with MS walked on the treadmill at their individual, sustainable speed while 20 healthy control subjects walked at all the different patient’s paces, allowing for comparisons independent of walking velocity. Kinematic analysis revealed pronounced restrictions in knee and ankle joint excursion, increased gait variability and asymmetry along with impaired dynamic stability in patients. The most discriminative single gait parameter, differentiating patients from controls with an accuracy of 83.3% (χ^2^ test; *p* = 0.0001), was reduced knee range of motion. Based on hierarchical cluster and principal component analysis, three principal pathological gait patterns were identified: a spastic-paretic, an ataxia-like, and an unstable gait. Follow-up assessments after 1 year indicated deterioration of walking function, particularly in patients with spastic-paretic gait patterns. Our findings suggest that impaired knee/ankle control is common in patients with MS. Personalised gait profiles and clustering algorithms may be promising tools for stratifying patients and to inform patient-tailored exercise programs. Responsive, objective outcome measures are important for monitoring disease progression and treatment effects in MS trials.

## Introduction

Multiple sclerosis (MS) is a chronic inflammatory disease of the CNS leading to demyelination of fibres and neuronal degeneration. MS is the leading cause of progressive functional impairment in young adults^1^. Three of four people with MS report reduced mobility due to impaired walking function at some point during their lifetime^2,3^. Walking impairment is perceived as the most restricting of symptoms by patients and constrains quality of life^4–6^.

Walking abnormalities in patients with MS are, nevertheless, poorly characterised. MS-related lesions may be widely disseminated throughout the brain and spinal cord, leading to a variety of gait abnormalities including ataxia, muscular weakness and spasticity-related signs^7–9^. Accordingly, there is no widely-accepted, typical gait pattern in MS unlike in other neurological disorders such as Parkinson’s disease or after unilateral cerebral stroke. Previous studies assessing gait deviations in patients with MS described reduced gait speed and step length^7,9–14^, reduced range of motion (ROM) of leg joints^7,12,15,16^, increased double-limb support^7,10,17^, and reduced dynamic stability^17^. In the aforementioned studies, however, patients and healthy controls walked at their respective self-selected speed, resulting in significant speed differences between groups. As the vast majority of locomotor parameters is strongly influenced by gait velocity^18^, slower walking speed in patients relative to controls is a major confounder, limiting the characterisation of MS-related gait pathophysiology^9,19,20^.

While several studies performed cross-sectional characterisation of walking dysfunctions in patients with MS, longitudinal assessments of the decline in walking performance in patients are rare^21–23^. However, monitoring of disease progression and walking dysfunction over time is important in the clinical setting, as it may help to adjust and optimise treatment strategies over the course of the disease^24^. Further, accumulative walking impairment is a hallmark of secondary progressive MS^25^ and changes in walking function based on detailed, reliable and quantitative assessments may serve as objective surrogate marker for conversion from relapsing-remitting to secondary-progressive MS. Comprehensive profiling of walking deficits, ranging from personalised gait signatures to objective cluster analysis of locomotor patterns, may help in monitoring therapies aimed at restoring walking function in patients and to identify sensitive endpoint measures for future trials in MS.

In this study, we characterised multiple sclerosis-related gait pathology, aimed at detecting and defining characteristic gait patterns in patients, and monitored deterioration of walking function over a period of one year.

## Results

### Study population

Thirty-seven patients with MS (48.6 ± 10.3 years; 24 female) and 20 able-bodied, healthy controls (48.8 ± 10.1 years; 12 female) were included (Table 1). Eleven of 37 patients used walking aids at least occasionally during everyday life. Demographic data of patients were not different from healthy subjects (Table 1). Ambulatory performance in clinical walking tests including maximal walking speed in the T25FW and endurance in the 6MWT were significantly reduced in patients (T25FW: −72%; 6MWT: −44%; *p* < 0.0001 for both tests; two-tailed, unpaired t-test; Table 1).

**Table 1:** Demographic data of patients and healthy control subjects. For statistical analysis, two-tailed, unpaired t-test or Fisher’s exact test was used. Abbreviations: EDSS: Expanded Disability Status Scale; DMT: disease-modifying treatment; PPMS: primary progressive MS; RRMS: relapsing remitting MS; SPMS: secondary progressive MS; s: strongest leg; T25FW: Timed-25 foot walk; w- weakest leg; 6MWT: 6-minute walk test.

	patients	healthy controls	P-values
**number of participants**	37	20	
**age [y]**	48.6 ± 10.3	48.8 ± 10.1	0.9286
**female [%]**	24/37	12/20	0.7780
**height [cm]**	170.4 ± 9.9	170.9 ± 7.0	0.4642
**weight [kg]**	68.5 ± 17.1	71.7 ± 11.5	0.8600
**disease type:** RRMS/PPMS/SPMS	22/2/13		
**disease duration [y]**	10.7 ± 6.9		
**T25FW [s]**	6.2 ± 2.7	3.6 ± 0.4	<0.0001
**6mWT [m]**	397.0 ± 116.0	709.9 ± 74.7	<0.0001
**EDSS [pts]**	4.5 ± 1.0		
**manual muscle testing:**			
hip flexion (w/s)	4.39/4.69	5/5	
knee flexion (w/s)	4.39/4.87	5/5	
knee extension (w/s)	4.74/4.93	5/5	
ankle extension (w/s)	4.47/4.87	5/5	
**DMT of multiple sclerosis** (number of patients)			
interferon	6		
fingolimod	4		
natalizumab	12		
no treatment	15		

### Impact of walking speed on kinematic gait parameters

v_max50_ was higher in healthy controls (1.08 ± 0.12 m/s) than in patients (0.64 ± 0.17 m/s; *p* < 0.0001, two-tailed, unpaired t-test; Supplementary Table S1). As previously reported by others^18,20,26^, we found that most kinematic gait parameters changed significantly with walking speed (Supplementary Table S1) demonstrating that differential walking speeds in patients and healthy controls confound the precise characterisation of walking deficits.

### Characterisation of walking impairments in patients with MS

**Figure 1:**
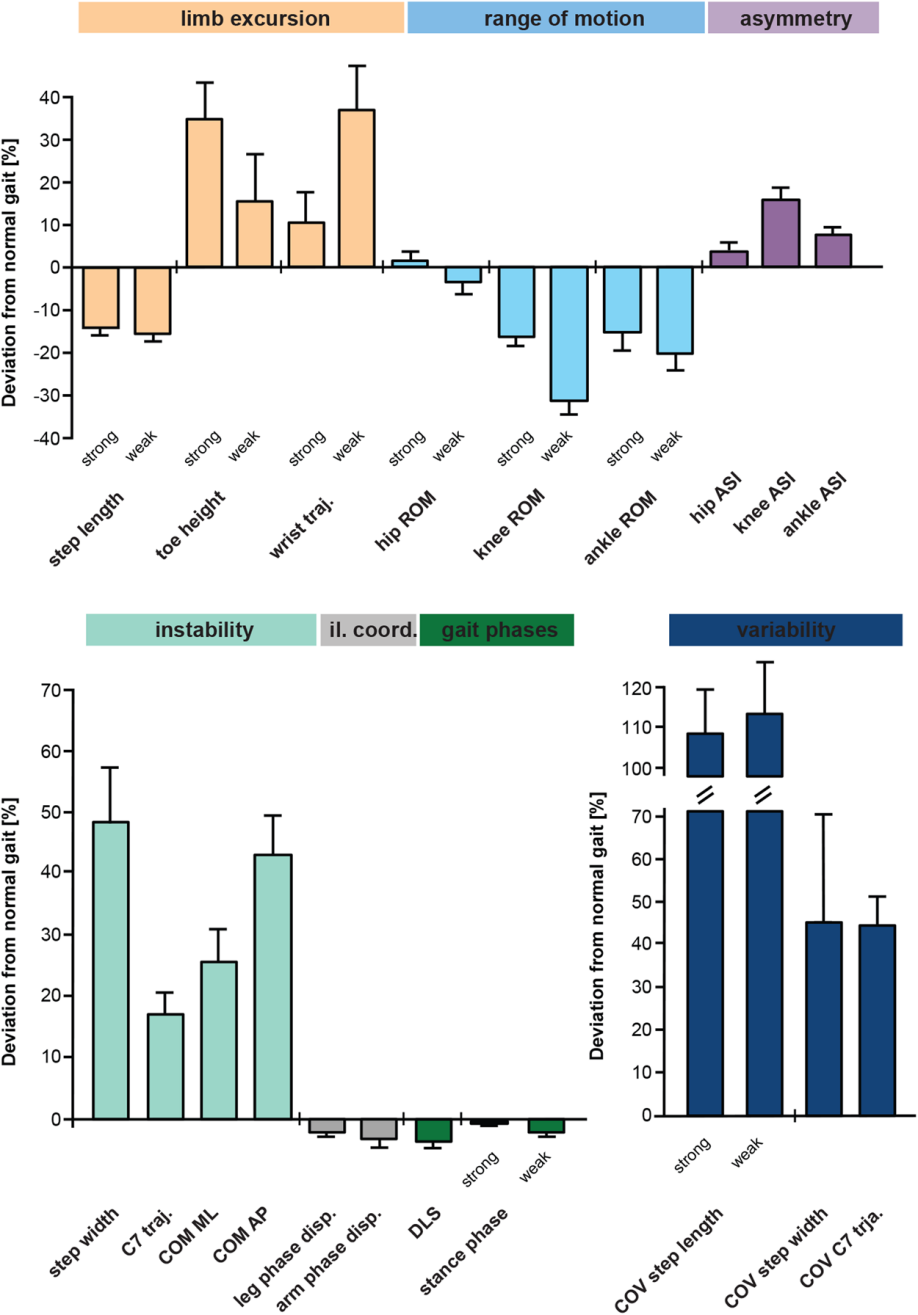
Characterisation of MS-related gait pathology based on 28 key walking parameters. Gait markers of limb excursion (salmon pink), parameters of range of motion (blue), asymmetry (purple), stability (turquoise) and variability (dark blue) revealed substantial changes of walking pattern in patients with MS (n = 37) walking at half-maximal gait speed (v_max50%_) compared to normative data obtained from 20 healthy control participants walking at identical speeds. Bars represent mean values ± SEM. Abbreviations: ASI: asymmetry index; AP: anteroposterior; COM: centre of mass; COV: coefficient of variation; disp.: dispersion; DLS: double-limb support; il. coord.: inter-limb coordination; strong: strongest leg; weak: weakest leg; ML: mediolateral; ROM: range of motion; traj.: trajectory.

Patients showed a bilateral reduction in step length, which was associated with diminished ROM in the leg joints (Fig. 1). While ROM of the hip was preserved, significant reductions in the extent of movement were observed at the knee in particular, but also at the ankle joint. The more pronounced restriction of ROM at the knee and ankle joint of the weakest leg resulted in substantial left-right asymmetry. Mid-swing toe clearance in the strongest leg was increased, while the length of the wrist movement trajectory ipsilateral to the weakest leg was increased by more than 30% (Fig. 1). Most markers of dynamic instability such as step width (+49%), upper trunk movement (C7 trajectory: +17%) and the mediolateral (+25%) and anteroposterior (+43%) components of the centre of mass (COM) trajectory were increased in patients. Gait variability was strongly increased in patients while parameters of inter-limb coordination and stance phase duration only showed minor deviations in patients (Fig. 1 and Supplementary Table S1).

**Figure 2:**
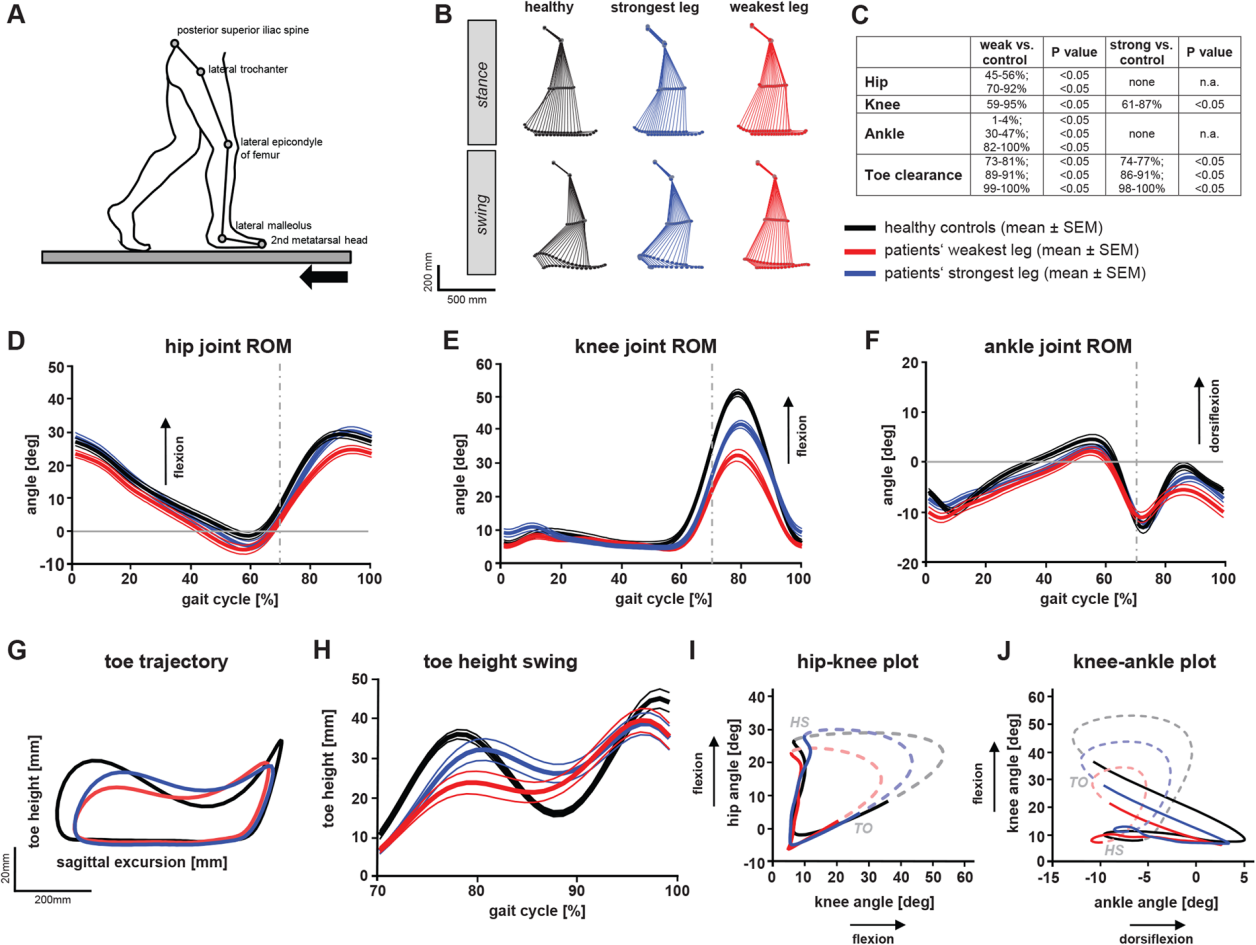
Temporal and spatial deviations in gait pattern of patients with MS compared to controls. Both patients and controls walked at a fixed speed of 2 km/h. **(A)** Schematic illustration of stick figure and marker position overlying anatomical landmarks. **(B)** Average stick figures of healthy participants (n = 20; black) and patients (n = 28; strongest leg (blue); weakest leg (red)) during stance and swing phase. **(C)** Statistical analysis (two-way ANOVA repeated measures) depicting periods (gait phases; left) and *p*-values (right) of significant differences between angular hip, knee and ankle excursions of patients (strongest and weakest leg) and healthy controls (see **D**–**F**). (**D**–**F**) Hip, knee and ankle angular excursions ± SEM during an averaged step cycle (groups and colors as in B; grey dashed line indicates toe-off). **(G)** 2-dimensional toe trajectory illustrating the sagittal movement pattern of the lower extremity endpoint (groups and colors as in B). **(H)** Toe clearance during swing phase (groups and colors as in B). **(I, J)** Angle-angle plots (cyclograms) depicting intra-limb coordination of the hip, knee and ankle during stance (straight line) and swing phase (dashed line) (groups and colors as in B). Abbreviations: deg: degrees; strong: strongest leg; weak: weakest leg; ROM: range of motion.

We also investigated curve progressions of leg joint angular excursions over the entire gait cycle in controls (n = 20) and patients (n = 28) walking at a fixed speed of 2 km/h (Fig. 2). Hip angles of the patients’ weakest leg revealed a subtle shift of 5 degrees towards extension (Fig. 2C,D). Stick figures and angle plots demonstrated the reduced knee flexion in patients, mainly in the weakest leg (red) during swing phase (Fig. 2B–E). Angular excursion of the ankle joint showed a decrease in ankle dorsiflexion of the weakest leg during stance and swing phase (Fig. 2C,F). Analysis of leg end-point control (toe trajectory) revealed reduced and delayed toe clearance in patients during early swing phase (Fig. 2G,H), possibly due to the delayed initiation of knee flexion at pre-swing (Fig. 2E). Patients’ toe height was increased during mid-swing but reduced at heel-strike, the latter indicating impaired foot roll dynamics (Fig. 2F–H). Intra-limb joint coordination revealed similar cyclogram shapes in patients and controls indicating preserved temporal coupling of leg joints (Fig. 2I,J). Differences in the size of the cyclograms were mainly due to pathological changes in knee and ankle ROM.

Binary logistic regression analysis of kinematic data (Fig. 2) revealed that the best indicator of pathological gait was knee ROM of the weakest leg, achieving an accuracy of 83.3% in classifying patients with MS and healthy participants (χ^2^ test: *p* = 0.0001).

### Individual gait signatures in patients with MS

**Figure 3:**
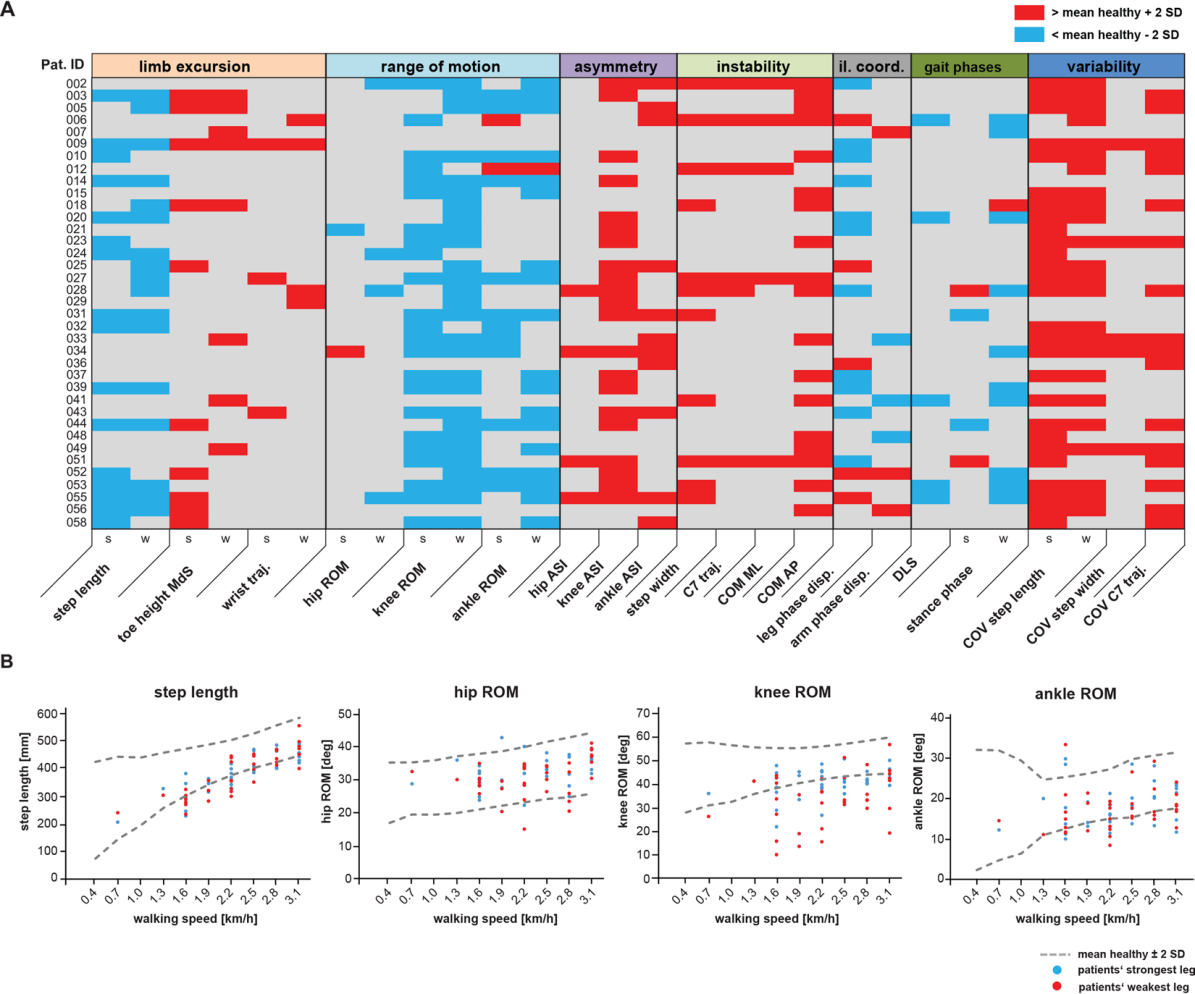
Individual gait profiles of patients with MS. **(A)** Kinematic gait parameters for single subjects with MS (n = 37) walking at half-maximal gait speed (v_max50%_) represented in a color-coded grading system. Twenty-eight parameters were classified as normal/unchanged (grey), pathologically reduced (blue) or pathologically increased (red) based on normative data obtained from 20 healthy controls. **(B)** Graphs show changes in selected gait parameters in subjects with MS in relation to walking speed. Values located outside the interval of the mean ± 2 SD of 20 healthy control subjects (dashed grey line) were defined as pathological. Data for the weakest leg is displayed in red, while values for the strongest leg are shown in blue. Abbreviations: AP: anteroposterior; ASI: asymmetry index; COM: centre of mass; COV: coefficient of variation; disp.: dispersion; deg: degrees; DLS: double-limb support; il. coord.: inter-limb coordination; s: strongest leg; MdS: midswing; ML: mediolateral; Pat. ID: patient identification number; ROM: range of motion; SD: standard deviation; traj.: trajectory; w- weakest leg.

Detailed gait profiles were generated for each patient to uncover individual deficits of walking function (Fig. 3). At each walking speed and parameter, the mean value ± 2 standard deviations measured in 20 control subjects was defined as the normal range (Fig. 3B). Values above or below this range were defined as pathological (Fig. 3A). The general distribution of the individual, pathological changes reflected the findings at the group-level (Figs 1, 2), for example, reduced step length and ROM of the more distal leg joints (Fig. 3A). Individual gait profiles also exposed a degree of heterogeneity in walking patterns, for instance, patients with pronounced reductions in distal leg joint excursions (014) or with a more unstable and variable walking pattern (023).

### Classification of predominant walking patterns in MS

**Figure 4:**
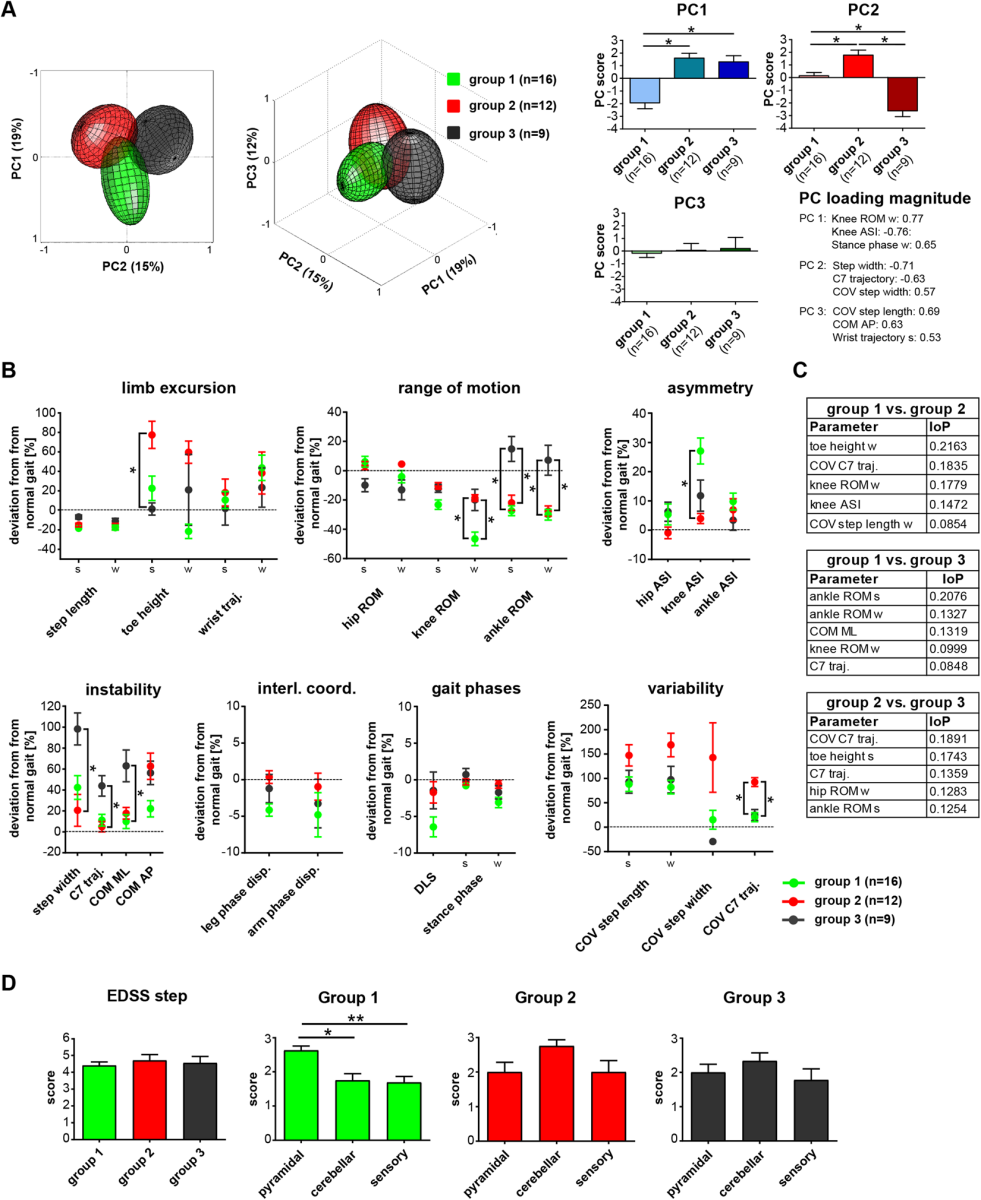
Kinematic gait patterns of three subgroups of patients with MS as identified by hierarchical cluster analysis. Gait parameters were assessed at patients’ half-maximal walking speed (v_max50%_) **(A)** Principal component analysis (PCA) visualizing the locomotor pattern of the three cluster subgroups (group 1: n = 16; green; group 2: n = 12; red; group 3: n = 9; black) in the 3-dimensional PC space. Graphs on the right show mean scores on PC1–3 for all three cluster subgroups. Asterisk indicates statistically significant differences (*p* < 0.05) between PCs for different cluster subgroups as revealed by 1-way ANOVA repeated measures with post hoc correction for multiple testing. Variables showing the highest factor loading on PC1, PC2 and PC3 (i.e. correlation between each variable and PC space) are depicted (right low corner). **(B)** Gait profiles of the three cluster subgroups based on single kinematic parameters. Dots represent group mean values ± SEM for each cluster subgroup. Asterisk indicates statistically significant differences between subgroups as analysed by 1-way ANOVA repeated measures followed by *post hoc* correction for multiple comparisons (*p < 0.05/28 = 0.0018). **(C)** ReliefF feature selection algorithm was used to quantify the importance of predictors (IoP) to distinguish the different cluster groups of specific kinematic parameters **(D)** Expanded Disability Status Scale scores for the different cluster subgroups (group 1: green; group 2: red; group 3: black). Asterisk indicates statistically significant differences between groups as revealed by 1-way ANOVA repeated measures (*p < 0.05; **p < 0.01). Data are presented as mean ± SEM. Abbreviations: AP: anteroposterior; ASI: asymmetry index; COM: centre of mass; COV: coefficient of variation; disp.: dispersion; DLS: double-limb support; EDSS: Expanded Disability Status Scale; interl.coord.: inter-limb coordination; s: strongest leg; ML: mediolateral; w- weakest leg; PC: principal component; ROM: range of motion; SD: standard deviation; traj.: trajectory.

Hierarchical cluster analysis was used to identify the main patterns of walking dysfunction in PwMS. In a first step, the clustering algorithm was validated with a dataset consisting of 28 gait parameters assessed in 20 healthy participants and 28 patients walking at 2 km/h. The algorithm demonstrated 93.8% accuracy in distinguishing between patients and healthy controls. We then used the same algorithm to identify the prevailing gait patterns in the main cohort of patients (n = 37) walking at v_max50%_ and identified three different gait patterns (Fig. 4). PCA revealed group data clouds that were largely separated in the PC space, thus confirming differential walking patterns of the three identified cluster subgroups (Fig. 4A). Subgroup 1 was characterised by a particularly marked reduction in knee ROM and increased left-right knee asymmetry, but little impairment in dynamic stability and gait variability. Subgroup 2 revealed increased toe height at mid-swing with strongly enhanced gait variability, but with relatively well-preserved walking stability and symmetry. In contrast, subgroup 3 demonstrated pronounced dynamic instability with less-impaired joint excursions, in particular at the ankle (Fig. 4B). ReliefF feature selection analysis confirmed the main relative discriminants identified in the cluster and PC analysis (Fig. 4C).

The EDSS step was not different between the three cluster subgroups (Fig. 4D; 1-way ANOVA). However, pyramidal dysfunction scores were higher in subgroup 1 (Fig. 4D; pyramidal vs. cerebellar: *p* = 0.0115; pyramidal vs. sensory: *p* = 0.0016; 1-way ANOVA, repeated measures), whereas subgroup 2 showed a trend towards more pronounced cerebellar deficits. Walking performance in clinical walking tests and muscle strength of the different leg muscle groups (LEMMT) were not different between the three cluster subgroups (Supplementary Fig. S1; 1-way ANOVA; 2-way ANOVA, repeated measures, respectively). Proportions of concomitant MS therapies were not different between the cluster groups (*p* = 0.656; χ^2^ test).

### Decline of waking function in patients with MS over 1 year

**Figure 5:**
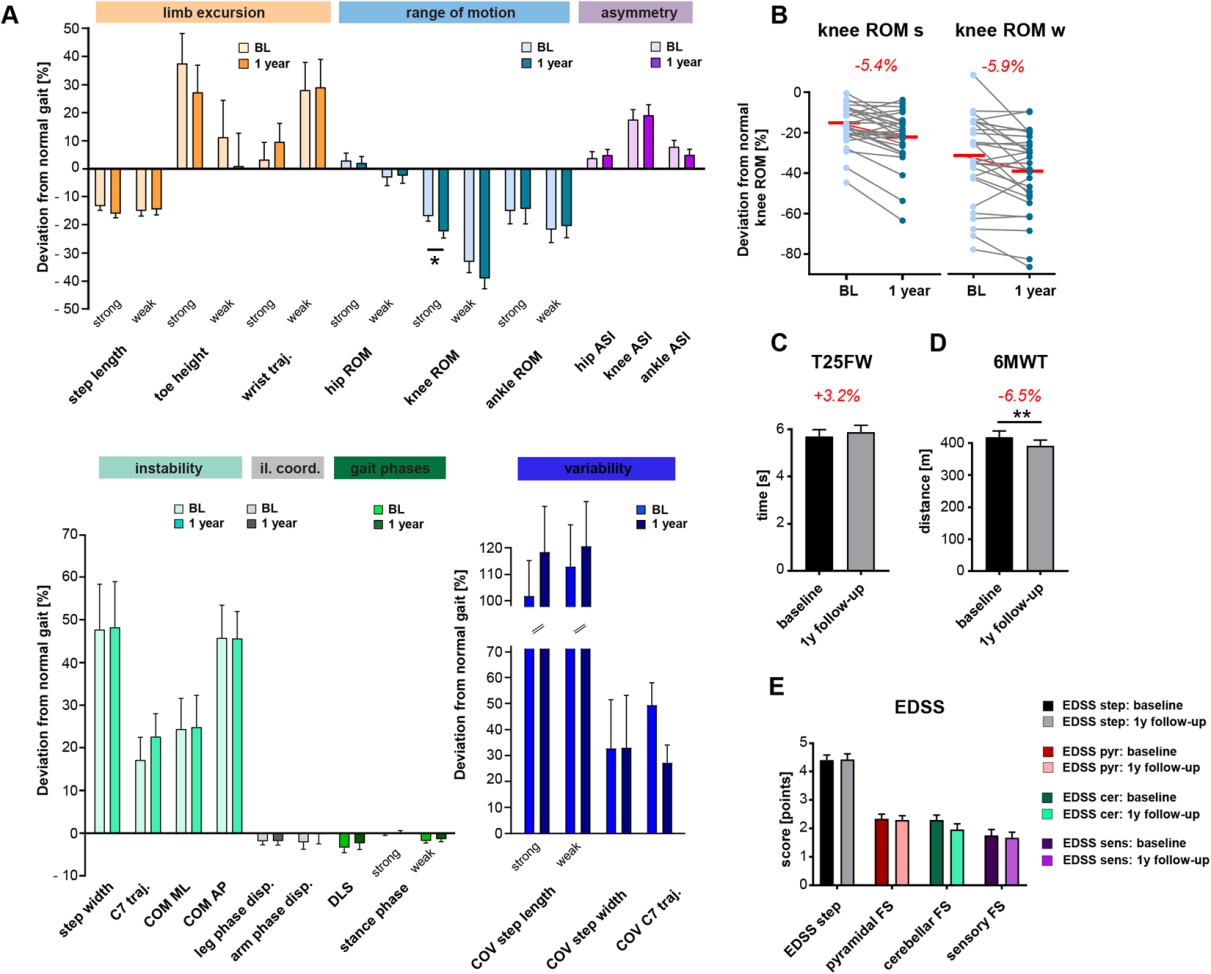
Monitoring kinematic gait parameters and clinical walking performance in patients with MS over a period of 1 year. Gait analysis in patients (n = 29) was performed at baseline and 12 months later at identical walking speeds (v_max50%_). **(A)** Gait profiles including 28 kinematic parameters with darker colors representing parameter values at baseline and brighter colors indicating values at the 1-year follow-up assessment. Bars represent mean ± SEM. Statistical analysis was performed by two-tailed, paired t-test followed by *post hoc* correction for multiple testing. Asterisk indicates *p*-value below the adjusted level of significance (α = 0.05/28). **(B)** Changes of individual patients’ knee ROM over 1 year for the weakest and strongest leg. Group mean changes in knee ROM between baseline and follow-up measurements are highlighted in red numbers. **(C)** Maximal walking speed (assessed with the timed 25-foot walk) was not significantly different, whereas **(D)** walking endurance (measured with the 6-minute walk test; 6MWT) showed a significant decline over time. Statistical significance was assessed by two-tailed, paired t-test. **(E)** Longitudinal assessment of the EDSS step and the functional system scores (pyramidal, cerebellar and sensory) did not reveal any significant differences over 1 year (two-tailed, paired t-test). Abbreviations: AP: anteroposterior; ASI: asymmetry index; COM: centre of mass; COV: coefficient of variation; disp.: dispersion; DLS: double-limb support; EDSS: Expanded Disability Status Scale; FS: functional system; cer: cerebellar; interl.coord.: inter-limb coordination; strong: strongest leg; ML: mediolateral; weak: weakest leg; pyr: pyramidal; ROM: range of motion; SD: standard deviation; sens: sensory; traj.: trajectory; T25FW: Timed 25-foot walk; v50%: half-maximal walking speed; y: year; 6MWT: 6-minute walk test.

Twenty-nine of 37 patients were available for follow-up assessment, with eight patients declining further participation in the study. Demographics of the remaining patients were statistically not different from the baseline cohort and the healthy control group (two-tailed, unpaired t-test or Fisher’s exact test). Two participants experienced a clinically-apparent relapse between baseline and follow-up assessments, but none were in an active relapse phase during measurements. Proportions of concomitant MS therapies were not different between the cluster groups (*p* = 0.935; χ^2^ test). Only 2 of 29 patients revealed changes in concomitant MS treatment over 1 year. Kinematic analysis after 1 year revealed subtle but significant differences in walking patterns over time. Most gait parameters remained similar over 1 year. The only significant change in gait kinematics concerned an increasing impairment in knee joint excursions (knee ROM of strongest leg; *p* = 0.0129; two-tailed, paired t-test; Fig. 5A). Knee ROM worsened in 21 of 29 patients, both in the weakest and strongest leg, resulting in a bilateral reduction of ROM of around 6% over a period of 1 year (Fig. 5B). Clinical walking tests demonstrated significantly diminished walking endurance in patients after 1 year (−6.5% or −27.1 m in the 6MWT; *p* = 0.0027; two-tailed, paired t-test; Fig. 5D). Walking decline was pronounced in cluster subgroup 1 (T25FW: *p* = 0.0473; 6MWT: *p* = 0.0258; 2-way ANOVA, repeated measures; Supplementary Fig. S1). Interestingly, deterioration in knee ROM over time correlated with the decline in walking function in the T25FW (R = 0.421; *p* = 0.023; Pearson correlation) and 6MWT (R = 0.407; *p* = 0.029). The total EDSS step and its functional system subscores did not change over 1 year in our patient cohort (Fig. 5E; two-tailed, paired t-tests).

## Discussion

We prospectively characterised gait deficits in a cohort of 37 patients with MS applying sensitive, state-of-the-art 3D-kinematic gait analysis during treadmill walking and classified these patients into three different subgroups based on their walking pattern. We demonstrate mild, but significant, deterioration of walking function over a period of 1 year. Locomotor profiles were supplemented by neurological examination and clinical, overground walking tests.

In contrast to most cross-sectional comparative studies in this field of research^7,12,17^, the present study performed gait analysis beyond standard spatio-temporal walking parameters and generated full-body gait profiles based on a comprehensive set of upper and lower extremity and trunk kinematic parameters. Previous key studies describing gait abnormalities in patients with MS used participants’ freely-chosen walking speeds, resulting in paces that were typically reduced by 30–40% in patients compared to healthy controls^7,9,11,12,16,27^. We confirm that most walking parameters scale with walking speed and, therefore, conclude that accurate comparison of walking patterns between patients and healthy controls critically requires identical walking speeds. Thus, heterogeneous walking speeds in prior studies investigating walking deficits in MS likely explain some inconsistent results^7,12,17^. However, assessing gait while allowing patients to walk at their preferred speed is widespread due to its relevance to everyday life and the different demands placed on maintaining body control at slower vs. faster speeds^28^. To solve this problem, we compared each patient walking at a sustainable, moderate speed with 20 speed-matched controls for a more conclusive characterisation of gait impairments in MS.

In line with previous reports, we found reduced knee and ankle joint excursions in subjects with MS^12,20^. In contrast to earlier reports, however, we demonstrated that hip joint excursions and inter- and intra-limb coordination were barely affected in patients. Basic human and animal research suggests that these latter functions are primarily controlled by spinal interneuron networks known as central pattern generators (CPGs). The CPG networks are modulated by supraspinal and sensory drive but can also act independently of descending input^29–32^. In contrast, the strongly-affected distal limb control, including knee and ankle movements, as well as fine adjustments in foot positioning during walking to ensure dynamic stability, is thought to rely more heavily on the integrity of cortical, cerebellar and brainstem systems with their long descending and ascending myelinated fibre tracts^33–35^. The pattern of functional impairment described above may be explained by the common sites of MS lesions and resulting axonal loss and neurodegeneration within the CNS of PwMS: lesions are less pronounced in spinal grey than white matter and there seems to be a predilection for involvement of the long descending and ascending fibre tracts in comparison to spinal interneuron circuits such as CPGs^36,37^. These concepts should be further investigated using magnetic resonance imaging to assess correlations between lesioned CNS structures and the resulting functional impairments. The walking pattern of patients with MS described here is different from other neurological conditions such as that seen in Parkinson’s disease^38^ or patients with incomplete spinal cord injury, with the latter showing increased hip angular excursion in the context of unaltered knee angular excursion compared to controls walking at the same speed^39^.

Of all gait parameters evaluated in this study, impaired knee flexion was one of the main kinematic features of MS gait reaching an accuracy of 83% in differentiating gait patterns of patients from controls^40^. This is in line with previous studies reporting that impaired knee flexion is an important MS-associated gait characteristic and a valid predictor of walking function/gait speed in these patients^41,42^. Intriguingly, improved knee flexion as induced by pharmacological agents (fampridine^40^, baclofen^43^), or with functional electrical stimulation^44^ or physical exercise^43,45^ have previously been associated with enhanced walking function in PwMS. Restoring knee excursion thus seems to be a promising approach for future treatment options aiming at improving walking function in PwMS^46^.

Using cluster and principal component analyses, we identified three subgroups in our patient cohort walking with distinctive gait patterns. The first subgroup was characterised by a pronounced reduction of knee ROM (particularly during swing phase), resulting in increased left-right asymmetry, and also decreased excursions in the ankle joints, while other aspects of walking function were largely preserved in these patients. This walking pattern may correspond most closely to a “spastic-paretic” gait as reported for patients with spastic hemi-/paraparesis^47,48^, in line with the clinical finding that these patients primarily presented with signs of pyramidal dysfunction. To what extent the reduced knee ROM during swing phase is caused by paresis (e.g. hamstrings), increase of muscle tone (e.g. rectus femoris) or decreased push-off power in the ankle joint (e.g. gastrocnemius) remains to be investigated in future studies^49^. Reduction of knee flexion strength in the weakest leg as assessed with BMRC manual muscle testing was not more pronounced for this subgroup of patients compared to cluster groups 2 and 3 (*p* = 0.8458). However, the ability of such static manual muscle testing to reliably reflect muscle function during dynamic conditions is disputed^50^. Reliable quantification of spasticity remains difficult and, therefore, was not considered in the present study^51–54^.

The most striking characteristic of the second subgroup was increased spatial variability of leg and trunk movements accompanied by increased toe height during mid-swing phase. Strongly increased gait variability is a cardinal feature in patients with sensory or cerebellar gait ataxia^55–60^. However, there was only a trend towards a more impaired cerebellar function in the clinical examination in these patients with an ataxia-like walking pattern. Increased toe height may serve as compensatory strategy to prevent tripping or falling in these patients^55,61^.

The third subgroup was mainly characterised by increased dynamic instability as evident by a broad-based gait and excessive trunk movements, both in the mediolateral and anteroposterior direction. Interestingly, these patients demonstrated increased ROM in their ankle joints, which may be considered a strategy to counteract impaired balance control^62^ or, in contrast, may reflect deficient ankle joint stabilization during walking. Postural instability appears to be a non-specific symptom that can be found in many neurological disorders including stroke, cerebellar disorders, traumatic brain injury and Parkinson’s disease^63–67^.

Stratifying patients with MS into different “functional” subgroups based on objective and comprehensive gait assessments may open new avenues for therapeutic approaches and may be important in the targeting of patient-tailored rehabilitative training programs. Also, disease progression may be different between subgroups; in our cohort, patients in subgroup 1 demonstrated the most marked deterioration of walking performance in the clinical walking tests over 1 year.

MS is a progressive disease and persisting functional deficits are thought to be primarily related to neurodegenerative processes during the advanced stages of the disease^68^. While the EDSS did not capture neurological decline in our cohort of patients, clinical walking tests and kinematic gait analysis demonstrated sufficient responsiveness to detect worsening of walking function over 1 year. Whereas changes in the T25FW (+3.2% or +0.18 s) were small and did not reach the threshold for clinically meaningful changes (≥20% improvement)^69,70^, deterioration in the 6MWT (−6.5% or −27.1 m) were significant and reached the threshold that has previously been associated with clinically meaningful changes in PwMS (>21.6 m)^71^. Walking deterioration in our cohort was slightly more pronounced when compared to an earlier report examining walking function of 109 PwMS over 2 years^23^. Specifically, gait analysis revealed deterioration of one key parameter of gait dysfunction in MS over time, namely ROM of the knee joint. The clinical relevance of this kinematic finding was supported by a correlated decline in performance in clinical walking tests. These results highlight the value of clinical walking tests as outcome measures for monitoring walking function and disease progression over time while also confirming the role of kinematic gait analyses in identifying the mechanisms underlying gait disturbances in MS^72^.

One limitation of the present study is that only patients able to walk without assistance on the treadmill were included, as the use of handrails during treadmill walking has a profound effect on gait patterns and obscures walking impairments^73^. The present results may therefore not be applicable to the full spectrum of MS-related gait disorders. However, one third of our patients used walking aids at least occasionally in everyday life, indicating that our population also included patients with notable walking deficits. Our data were performed during treadmill walking and thus might not completely reflect spontaneous, everyday, overground walking. However, a substantial amount of literature supports the conclusion that the basic biomechanics of treadmill walking are highly similar to overground walking given sufficient acclimatisation to treadmill walking prior to gait analysis^74–77^. Moreover, the sample size of this study is limited (mainly for measurements assessing walking deterioration) and results need to be confirmed in larger cohort studies.

To conclude, gait disturbances in a cohort of mildly- to moderately-affected patients with MS are typified by decreased knee and ankle flexion, reduced dynamic stability and increased variability of movements. Subgroup analysis highlighted three dominant walking patterns in these patients: spastic-paretic gait, ataxia-like gait and unstable gait. While sensitive kinematic gait analysis and clinical walking tests detected deterioration of walking function over 1 year, EDSS scores did not reveal any change in our cohort. These insights may help to inform rehabilitative gait training in patients and to develop deficit-specific and patient-tailored exercise programs.

## Materials

### Participants

Thirty-seven patients diagnosed with relapsing-remitting, primary- or secondary-progressive MS and clinical walking impairment were assessed at University Hospital Zurich between 2012 and 2013 (FAMPKIN study; clinicaltrials.gov, NCT01576354). For inclusion, patients had to be able to cover a distance of at least 50 meters within 6 minutes and to walk independently on an instrumented treadmill. Healthy control participants were measured within the framework of a two-centre study (University Hospital Zurich and University Hospital Balgrist). Both studies were approved by the Zurich cantonal ethics committee (KEK-2011-0445, KEK-2014-0004) and were conducted in accordance with the Declaration of Helsinki and Good Clinical Practice. All subjects gave written, informed consent.

### Study design and experimental procedures

All participants underwent a neurological examination including the Expanded Disability Status Scale (EDSS)^78^, the Lower Extremity Manual Muscle Test (LEMMT)^79^, the timed 25-foot walk (T25FW)^80^ and 6-minute walk test (6MWT)^81^. The maximal overground walking speed (v_max100%_) was determined from the T25FW and 50% of this speed (v_max50%_) was set as proportional walking speed on the treadmill for each patient. This approach allows direct comparison between patients with varying degrees of walking impairment and healthy controls and participants. The resulting treadmill speed is a moderate, sustainable walking velocity universally reported to be comfortable for treadmill walking by patients^82^. As such, we refer to v_max50%_ as a “sustainable” speed in this manuscript. Twenty healthy participants walked at different speeds ranging from 0.4 to 3.1 km/h (in intervals of 0.3 km/h) to match the individual walking speeds of all patients. All participants underwent an extensive acclimatisation protocol (≥15 minutes) to familiarise themselves with treadmill walking, as basic locomotor patterns on the treadmill approach spontaneous overground walking only in subjects well familiarised to treadmill walking^74,77,83^. Analysis of angular curves and cyclograms was conducted at a fixed speed of 2 km/h for all participants, as only an identical speed allows for a direct comparison/illustration of kinematic parameters in patients and controls. All other kinematic data of this paper are based on patients walking at their individual, sustainable speed (v_max50%_) as described above. In follow-up assessments after 12 months, patients again underwent kinematic gait analysis (while walking at v_max50%_) and clinical walking tests. All patients received treatment with prolonged-release fampridine within the framework of the FAMPKIN study^40,84^. However, treatment was discontinued 14 days before measurements.

Three-dimensional gait analysis was performed while participants walked barefoot for at least 30 seconds per trial on an instrumented treadmill (120 Hz, FDM-T, Zebris Medical GmbH, Germany) in a gait laboratory equipped with 14 infrared cameras recording via Nexus 2.2.3 (Vicon, Oxford, UK) motion capture software at a sampling rate of 200 Hz. Twenty-nine reflective markers (14 mm diameter) were placed on the skin based on the full-body gait model (Plug-in-Gait, Vicon, UK). Reflective markers were always placed by the same experienced physiotherapist and were visually inspected by a second team member, while the subject was in a standing position at full leg extension.

### Gait parameters and data analysis

Data were processed in Nexus 2.2.3 and parameters were extracted using ProCalc (Vicon, Oxford, UK) analysis software. Gait events were defined by zero-crossings in toe marker velocity as described previously^40,85^. The strongest and weakest leg of each patient was defined based on the neurological examinations. We quantified 28 key gait parameters describing leg, trunk and arm movements to comprehensively characterise walking patterns in patients and healthy controls. Gait parameters were assessed per step cycle and were classified into different functional domains: (1) limb excursion: step length, toe height at mid-swing phase, 3D path of wrist trajectory; (2) range of motion: range of motion (ROM) of hip, knee and ankle joints (leg joint angles were calculated by a 3D vector-based approach described elsewhere^86^; (3) asymmetry: left-right asymmetry of leg joint excursion as assessed by a modified asymmetry index (ASI; see equation (1)) used elsewhere^87,88^:

1 $${\rm{ASI}}=|(({\rm{left}}-{\rm{right}})/({\rm{\max }})\,({\rm{left}},{\rm{right}}))|\times \,100$$

(4) instability: step width, 3D path of cervical spine (C7) marker, centre of mass (COM) movements in mediolateral (ML) and anteroposterior (AP) directions; (5) inter-limb coordination: temporal left–right coordination of legs and arms as defined by phase dispersion^89,90^. In a subject walking perfectly in phase, heel strike of one limb occurs precisely at the mid-point of the contralateral step cycle, resulting in a phase dispersion of 0.5. (6) gait phases: stance phase, double-limb support (DLS); (7) variability: coefficient of variation (COV) of step length, step width and C7 marker trajectory.

### Statistical analysis

Statistical analysis was performed using SPSS (V23, SPSS Inc., CA, USA), Matlab (Mathworks, Inc., USA) and Graphpad Prism 7 (GraphPad Software, Inc., CA, USA). The applied parametric tests are described in the respective figure legends. The level of significance was set at 0.05. All statistical tests were adjusted for multiple comparisons via post-hoc Bonferroni correction. Personalised gait profiles (Fig. 3) were developed based on contrasts relative to the distribution of specific gait parameters in healthy subjects^91^. Binary logistic regression (Wald forward stepwise) was used to discover the principal gait parameters distinguishing gait patterns of patients from healthy control participants.

Hierarchical cluster analysis was performed using Ward’s method, described as particularly robust in identifying functional patterns^92^. Variables were z-standardised and the Euclidian distance used as measure of dissimilarity^93,94^. The optimal number of clusters was determined by the R ratio, a measure of intra-cluster variability. A threshold value of R > 15 was applied, similar to approaches used previously to identify differential patterns^94^. Key attributes classifying the different clusters were extracted using the RefliefF algorithm^95^. Walking data generated from the clusters analysis were examined by principal component analysis (PCA). Principal components were calculated using eigenvalue decomposition on the centred and standardised dataset. The number of relevant principal components was determined with a Kaiser-Guttman stopping criteria.

### Data availability

All data generated or analysed during this study are included in this published article (and its Supplementary Information files).

## Electronic supplementary material

Supplementary Information
