# Supplementary material for: Profiling walking dysfunction in multiple sclerosis: characterisation, classification and progression over time
Source: Sci Rep. 2018 Mar 21;8:4984. doi: 10.1038/s41598-018-22676-0 (PMC5862880; doi:10.1038/s41598-018-22676-0)
Supplement: Supplementary file 1 — Supplementary Information [file 41598_2018_22676_MOESM1_ESM.pdf]

Title:

Profiling walking dysfunction in multiple sclerosis: characterisation, classification and progression over time

Authors:

Linard Filli, PhD*†[1], Tabea Sutter, MD†[1], Christopher S. Easthope, PhD[2], Tim Killeen, PhD MRCS, Christian Meyer, MSc[2], Katja Reuter, MD[1], Lilla Lörincz, PT MSc[1], Marc Bolliger, PhD[2], Michael Weller, MD[1], Armin Curt, MD[2], Dominik Straumann, MD[1], Michael Linnebank, MD[1,3], Björn Zörner, MD PhD[1,2]

Affiliations: [1] Department of Neurology, University Hospital and University of Zurich, Frauenklinikstrasse 26, 8091, Zurich, Switzerland

[2] Spinal Cord Injury Center, Balgrist University Hospital, Forchstrasse 340, 8008 Zurich, Switzerland

[3] Department of Neurology, Helios-Klinik Hagen-Ambrock, / University Witten/ Herdecke, Ambrocker Weg 60, 58091 Hagen, Germany

* Corresponding author

† Authors contributed equally to the study

Corresponding author:

Linard Filli, PhD; Department of Neurology, University Hospital Zurich,

Frauenklinikstr. 26, 8091 Zurich, Switzerland; Phone: 0041 44 255 43 98,

Fax: 0041 44 255 43 80; E-Mail: linard.filli@usz.ch

Supplementary Table S1.

Characterization of gait dysfunctions in healthy controls and patients with multiple sclerosis at different walking speeds.

		absolute match of speed			relative match of speed		
		**controls**	**PwMS**	**Δ [%]**	**controls**	**PwMS**	**Δ [%]**
walking speed	m/s	0.64 ± 0.17	0.64 ± 0.17	0	1.08 ± 0.12	0.64 ± 0.17	-40.6
step length s	mm	449.6 ± 51.6	387.2 ± 73.4	-13.9	571.6 ± 42.5	387.2 ± 73.4	-32.3
step length w	mm	452.3 ± 55.5	383.8 ± 81.8	-15.1	580.7 ± 48.7	383.8 ± 81.8	-33.9
Toe Height MS s	mm	18.0 ± 1.1	24.1 ± 8.9	33.9	21.1 ± 4.3	24.1 ± 8.9	14.2
Toe Height MS w	mm	18.8 ± 1.1	21.9 ± 14.1	16.5	22.5 ± 6.0	21.9 ± 14.1	-2.7
Wrist trajectory s	mm	457.9 ± 105.5	500.3 ± 209.0	9.3	711.3 ± 203.8	500.3 ± 209.0	-29.7
Wrist trajectory w	mm	421.7 ± 101.7	555.4 ± 210.8	31.7	647.3 ± 198.5	555.4 ± 210.8	-14.2
Hip ROM s	deg	32.4 ± 2.3	32.7 ± 4.5	0.9	37.7 ± 5.8	32.7 ± 4.5	-13.2
Hip ROM w	deg	31.9 ± 2.1	30.8 ± 5.6	-3.4	37.0 ± 4.8	30.8 ± 5.6	-16.8
Knee ROM s	deg	49.2 ± 2.2	41.2 ± 6.0	-16.3	53.5 ± 3.1	41.2 ± 6.0	-23.0
Knee ROM w	deg	49.7 ± 2.1	34.4 ± 10.9	-30.8	54.5 ± 5.0	34.4 ± 10.9	-36.9
Ankle ROM s	deg	21.7 ± 2.1	18.4 ± 5.3	-15.2	26.6 ± 4.9	18.4 ± 5.29	-31.0
Ankle ROM w	deg	21.8 ± 2.2	17.4 ± 5.2	-20.2	26.9 ± 3.9	17.4 ± 5.2	-35.4
Hip ASI	n.a	8.3 ± 0.3	11.9 ± 11.4	3.6	7.8 ± 5.2	11.9 ± 11.4	4.1
Knee ASI	n.a	6.4 ± 0.2	22.3 ± 17.5	15.9	7.4 ± 5.1	22.3 ± 17.5	14.9
Ankle ASI	n.a	8.2 ± 0.7	15.5 ± 11.8	7.3	8.5 ± 6.7	15.5 ± 11.8	7.1
step width	mm	85.9 ± 2.5	127.6 ± 47.5	48.5	83.5 ± 22.3	127.6 ± 47.5	52.8
C7 trajectory	mm	171.6 ± 17.5	200.8 ± 50.0	17.0	163.5 ± 38.1	200.8 ± 50.0	22.8
COM ML	mm	37.7 ± 6.8	47.2 ± 16.7	25.2	27.7 ± 8.6	47.2 ± 16.7	70.2
COM AP	mm	24.1 ± 3.0	34.4 ± 9.8	42.7	19.9 ± 3.8	34.4 ± 9.8	72.7
leg phase disp.	%	49.9 ± 0.1	48.9 ± 2.2	-2.0	49.8 ± 0.6	48.9 ± 2.2	-1.9
arm phase disp.	%	49.3 ± 1.3	47.7 ± 5.0	-3.2	51.0 ± 2.1	47.7 ± 5.0	-6.5
DLS	%	39.5 ± 4.7	38.0 ± 4.9	-3.8	32.1 ± 2.1	38.0 ± 4.9	18.4
stance phase s	%	69.8 ± 2.4	69.6 ± 2.4	-0.3	66.0 ± 1.6	69.6 ± 2.4	5.4
stance phase w	%	69.7 ± 2.4	68.3 ± 2.8	-2.0	66.1 ± 1.0	68.3 ± 2.8	3.4
COV step length s	%	3.3 ± 0.9	6.9 ± 2.9	109.1	1.8 ± 0.5	6.9 ± 2.9	273.9
COV step length w	%	3.4 ± 0.9	7.5 ± 3.6	120.6	1.9 ± 0.5	7.5 ± 3.6	293.7
COV step width	%	22.5 ± 3.1	32.6 ± 35.0	44.9	22.8 ± 8.9	32.6 ± 35.0	40.2
COV C7	%	7.2 ± 0.5	10.7 ± 3.7	48.6	5.4 ± 1.5	10.7 ± 3.7	97.2

Abbreviations: ASI: asymmetry index; AP: anteroposterior; COM: centre of mass; COV: coefficient of variation; disp.: dispersion; DLS: double-limb support; il. coord.: inter-limb coordination; s: strongest leg; w: weakest leg; ML: mediolateral; ROM: range of motion; traj.: trajectory.

Supplementary Figure S1.

Demographics and clinical characteristics of cluster subgroups.

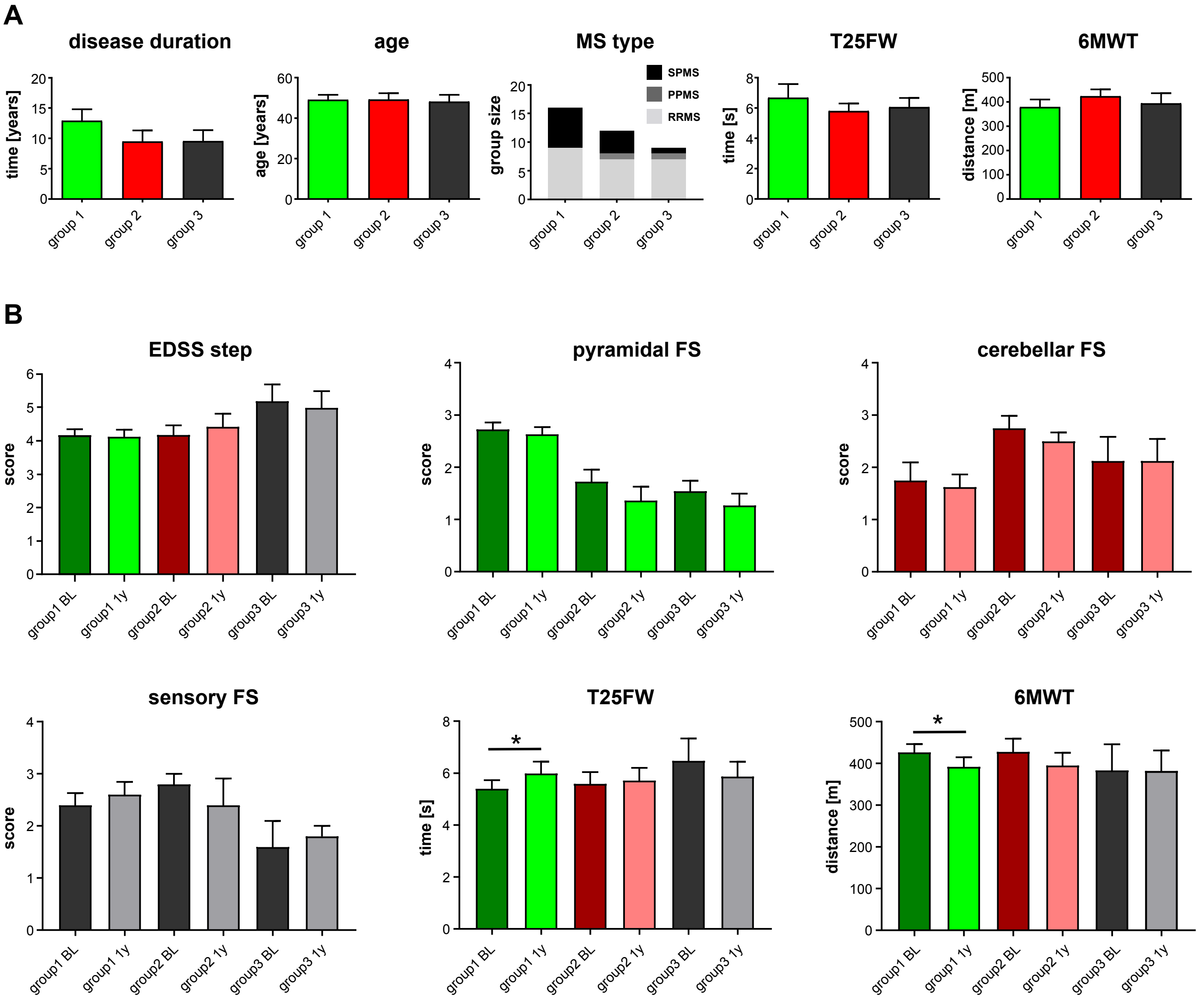

(A) Baseline characteristics of patients belonging to cluster subgroup 1 (n=16; green), subgroup 2 (n=12; red) and subgroup 3 (n=9; dark grey). There were no statistical differences between cluster subgroups (1-way ANOVA and Fisher's exact test). (B) Longitudinal analysis of clinical readouts for each cluster subgroup (subgroup 1 n=13, green; subgroup 2 n=10, red; subgroup 3 n=6, grey) over 1 year. For all subgroups, neurological function (Expanded Disability Status Scale and functional system scores) and maximal walking speed (assessed by T25FW) did not reveal any significant changes over time. Walking endurance (assessed by 6MWT) was significantly reduced in cluster subgroup 1, but not in subgroup 2 or 3. Statistical analyses of longitudinal data were performed by two-tailed, paired t-tests followed by post hoc correction for multiple testing. Abbreviations: BL: baseline; cer: cerebellar; EDSS: Expanded Disability Status Scale; FS: functional system; PPMS: primary progressive MS; pyr: pyramidal; RRMS: relapsing remitting MS; SPMS: secondary progressive MS; sens: sensory; T25FW: timed 25-foot walk; 1y: 1 year; 6MWT: 6-minute walk test.
